# Reduced level of physical activity during COVID-19 pandemic is associated with depression and anxiety levels: an internet-based survey

**DOI:** 10.1186/s12889-021-10470-z

**Published:** 2021-03-01

**Authors:** Paulo José Puccinelli, Taline Santos da Costa, Aldo Seffrin, Claudio Andre Barbosa de Lira, Rodrigo Luiz Vancini, Pantelis T. Nikolaidis, Beat Knechtle, Thomas Rosemann, Lee Hill, Marilia Santos Andrade

**Affiliations:** 1 Department of Physiology, Federal University of São Paulo, São Paulo, Brazil; 2 Human and Exercise Physiology Division, Faculty of Physical Education and Dance, Federal University of Goiás, Goiânia, Brazil; 3 Center for Physical Education and Sports, Federal University of Espírito Santo, Victoria, Brazil; 4 School of Health and Caring Sciences, University of West Attica, Athens, Greece; 5 Medbase St. Gallen Am Vadianplatz, St. Gallen, Switzerland; 6 Institute of Primary Care, University of Zurich, Zurich, Switzerland; 7 Divison of Gastroenterology and Nutrition, Department of Pediatrics, McMaster University, Hamilton, Canada

**Keywords:** Pandemic, Social distancing, COVID-19, Physical exercise, Mood state, Depression, Anxiety

## Abstract

**Background:**

The coronavirus disease 2019 (COVID-19) pandemic has resulted in a strong negative impact on economic and social life worldwide. It has also negatively influenced people’s general health and quality of life***.*** The aim of the present study was to study the impact of social distancing on physical activity level, and the association between mood state (depression and anxiety level) or sex with actual physical activity levels, the change in physical activity caused by social distancing period, the adhesion level to social distancing, the adoption time of social distancing, family income and age.

**Methods:**

A self-administered questionnaire with personal, quarantine, physical activity, and mood state disorders information’s was answered by 2140 Brazilians of both sex who were recruited through online advertising.

**Results:**

The physical activity level adopted during the period of social distancing (3.5 ± 0.8) was lower than that the adopted prior to the pandemic period (2.9 ± 1.1, *p* < 0.001). Thirty percent of the participants presented symptoms of moderate/severe depression and 23.3% displayed moderate/severe anxiety symptoms. A greater presence of symptoms related to anxiety and depression were associated with low physical activity levels, low family monthly income, and younger age. A higher percentage of men who had no mood disorders was observed among those who were very active than among those less active.

**Conclusion:**

The COVID-19 pandemic has a negative impact on physical activity. Those who reduced their level of physical activity had the highest levels of mood disorders. Therefore, physical activity programs should be encouraged, while respecting the necessary social distancing to prevent the spread of Severe Acute Respiratory Syndrome Coronavirus 2.

## Background

The coronavirus disease 2019 (COVID-19) pandemic caused by Severe Acute Respiratory Syndrome Coronavirus 2 (SARS-CoV-2) raised several questions about public health, economic, and political crisis [1].

After the initial outbreak, which occurred in December 2019 in Wuhan, China [2], the virus rapidly spread across China and reached Europe and both Americas [3, 4] and finally across the world. The first recorded case in Latin America was in São Paulo, Brazil, on February 26, 2020 [5]. In the following month (March 2020), several measures to control and contain the virus spread were taken by government institutions and health authorities in Brazil [6]. In the second half of March, schools and parks were closed and, commercial activities and non-essential services were suspended [6]. All these measures were taken in order to implement physical distancing among people to contain the spread of SARS-CoV-2, which has been considered a fundamental method to contain the virus spread [7]. As a consequence, these measurements of social distancing also may negatively impact the daily physical activity of the population [7–13].

Physical inactivity has been considered a global pandemic since 2012 [14], and it is estimated that 28% of the world population (1.4 billion people) remain physically inactive [15]. This scenario is extremely worrying because physical inactivity is one of the leading causes of cardiovascular diseases, diabetes, obesity, and premature mortality in the world [15–17]. Therefore, if the population’s physical activity levels further decrease during this physical distancing period, it will be an even greater challenge for public health agencies, as this condition may further complicate the pandemic scenario since the presence of diabetes, obesity, hypertension, and other comorbidities associated with physical inactivity can worsen the COVID-19 prognosis [7, 17].

In addition, physical distancing/isolation measures and the continuous spread of the pandemic are also expected to influence the mental health of the population [18]. Excessive information, uncertainties regarding the future and one’s health, frustration due to interruption of projects, boredom, reduction of family income, as well as a political and economic crisis, can generate or exacerbate symptoms of depression and anxiety levels [19, 20]. These problems associated with low levels of physical activity may also negatively impact mental health.

This scenario of physical inactivity and physical distancing/isolation measures may exacerbate existing health issues and social inequities for the female population [21, 22]. Descriptive studies reveal that women have a significantly higher risk than men for developing anxiety and depression disorders [23–25]. Moreover, some studies have reported that women consistently have lower physical activity levels than men [26, 27]. However, the effects of physical distancing on physical activity levels between the different sexes are still unknown. It is reasonable to assume that the impact on physical activity levels would be greater in women, as they accumulate professional tasks with household tasks such as taking care of children now the schools are closed [28].

### Study aims

#### The aims of this study are as follows

1. To study the impact of social distancing on physical activity level.
2. To study the association between mood state (depression and anxiety level) with current physical activity levels, the change in physical activity levels in relation to those prior to social distancing, the adhesion level to social distancing, the adoption time of social distancing, total family income and age in a sample of the Brazilian population.
3. To study the association level between depression and anxiety level.
4. To study the association level between sex with mood state (depression and anxiety level), current physical activity levels, the change in physical activity levels in relation to those prior to social distancing, the adhesion level to social distancing, and total family income in a sample of the Brazilian population.

## Methodology

### Study design

This was a cross-sectional study, which used a questionnaire to gather data for the study. The questionnaire was structured and shared using the digital platform; Google Forms and data was collected between June 02 and June 12, 2020. At the time of the survey the Brazilian government adopted emergency measures nationwide, including closure of schools and universities, parks, commercial activities and non-essential services but not lockdown. The questionnaire was self-administered in Portuguese language and contained five sections as described below.

### Questionnaire

The first section dealt with general data regarding the participant demographics. It contained questions related to sex (men or women), age in years (open-ended question), body mass in kg (open-ended question), height in cm (open-ended question), total family income measured in multiples of the minimum wage (less than 1 minimum wage, minimum wage between 1 and 2, minimum wage between 3 and 6, minimum wage between 7 and 10, more than 11 minimum wages). A minimum wage corresponds to less than $200 US per month according to the exchange rate of June 2020. For analysis purposes, scores from 0 to 4 were assigned to total family income, where 0 referred to the lowest income (less than 1 minimal wage), 1 referred to minimum wage between 1 and 2, 2 referred to minimum wage between 3 and 6, 3 referred to minimum wage between 7 and 10, and 4 referred to more than 11 minimum wages.

The second section contained questions related to behaviour during quarantine. This included an individual’s level of restriction specifically pertaining to routine activities (taking complete measures of social distancing and did not go out to perform any activity, leaving only for essential non-work activities, such as buying food, medicine or going to the doctor, leaving only for essential activities including work activities, and not taking any measures of physical distancing). For analysis purposes, scores from 0 to 3 were assigned to an individual’s level of restriction, where 0 referred to the higher restriction level and 3 to the lower restriction level. The second section also contained questions of how many days he or she adopted the physical distancing measures (less than 30 days, between 30 and 45 days, between 46 and 60 days, between 61 and 75 days, between 76 and 90 days, more than 91 days). For analysis purposes, scores from 0 to 5 were assigned to the duration of the social distancing measurements adopted, where 0 referred to the lower duration (less than 30 days) and 5 to the higher duration (more than 91 days).

The third section was dedicated to assessing the volunteers’ current physical activity level. To this end, the International Physical Activity Questionnaire (IPAQ) proposed by the World Health Organization in 1998 was used [29]. This instrument has acceptable measurement properties for estimating physical activity levels with previously reported internationally validated results [29] and was validated for the Portuguese language in 2001 [30, 31]. According to the answers provided by the participants, the level of physical activity was classified into 5 categories according to Matsudo et al. [31]: very active (those who perform vigorous activities 5 days/week and ≥ 30 min per session or vigorous activities ≥3 days/week and ≥ 20 min per session + moderate activities ≥5 days/week and ≥ 30 min per session), active (those who perform vigorous activities ≥3 days/week and ≥ 20 min per session; or moderate activities ≥5 days/week and ≥ 30 min per session; or any combined activity: ≥5 days/week and ≥ 150 min/week such as walking + moderate + vigorous), irregularly active A (those who perform physical activities but it is insufficient to be classified as active because it does not comply with the recommendations regarding frequency or duration), irregularly active B (those who perform physical activity but it is insufficient to be classified as irregularly active A because it does not comply with either the frequency or duration recommendations), not active (those who do not perform any physical activity for at least 10 continuous minutes during the week). For the purpose of analysis, scores from 0 to 4 were assigned to activity levels, where 0 referred to the lowest level of activity (not active) and 4 to the highest level of activity (very active).

The fourth section aimed to screen for possible mood disorders. The Patient Health Questionnaire-9 (PHQ-9) and General Anxiety Disorder-7 (GAD-7) questionnaire were applied. PHQ-9 is an instrument, validated for Portuguese, which is widely used to identify individuals at risk of depression [32, 33]. The questionnaire provides a final score ranging from 0 to 27. Scores of ≤4 suggest minimal depression, scores from 5 to 9 suggest mild depression, scores from 10 to 14 suggest moderate depression, scores from 15 to 19 suggest moderately severe depression, and scores of 20 or greater suggest severe depression. For the purpose of analysis, scores from 0 to 4 were assigned to the levels of depression. Scores of ≤4 (minimal depression) represented 0, scores from 5 to 9 (mild depression) represented 1, scores from 10 to 14 (moderate depression) represented 2, scores from 15 to 19 (moderately severe depression) represented 3 and scores of 20 or greater (severe depression) represented 4. GAD-7 aims to identify possible generalized anxiety disorders and also has a validated Portuguese version [34, 35]. The questionnaire provides a final score ranging from 0 to 21. Scores of ≤4 suggest no anxiety disorder, scores from 5 to 9 suggest mild anxiety, scores from 10 to 14 suggest moderate anxiety and scores of 15 or greater suggest severe anxiety disorder. For the purpose of analysis, scores from 0 to 3 were assigned to the anxiety levels. Scores of ≤4 (no anxiety disorder) represented 0, scores from 5 to 9 (mild anxiety) represented 1, scores from 10 to 14 (moderate anxiety) represented 2, scores of 15 or greater (severe anxiety) represented 3.

The last section again used the IPAQ questionnaire to assess physical activity. However, unlike the third section, the questions concerned the exercise routine in the period prior to quarantine and the recommended social distancing measures (prior to March, 2020). To analyze the effect of physicaldistancing on the level of physical activity, the difference in the level of physical activity was calculated as the IPAQ score obtained in the current condition minus the score obtained according to the condition before the period of physicaldistancing (ΔIPAQ). For analysis purposes, scores from − 1 to 1 were assigned to the difference in the level of physical activity between current and previous pandemic level, where − 1 referred to a reduction in the physical activity level, 0 referred to no difference in physical activity level, and 1 referred to an increase in physical activity level.

### Participants

Participants were invited to partake in the study through websites, e-mail, and social networks (Instagram, Facebook, and Whatsapp) of the researchers and institutions involved. The invitation contained a link to access the questionnaire, shared through the Google Forms digital platform. No incentives were used in this survey.

The inclusion criteria was over 18 years of age. Individuals from 26 Brazilian states and the Federal District answered the questionnaire. Body mass index (BMI) was calculated [weight in kilograms (kg)/ height in metres squared (m^2^)]. If BMI was less than 18.5, it was classified as underweight (category 1), between 18.5 to < 25, as healthy range (category 2), between 25 to < 30, as overweight (category 3), between 31 to < 35, as obese (category 4) and higher than 35, as extremerly obese (category 5).

**Fig. 1:**
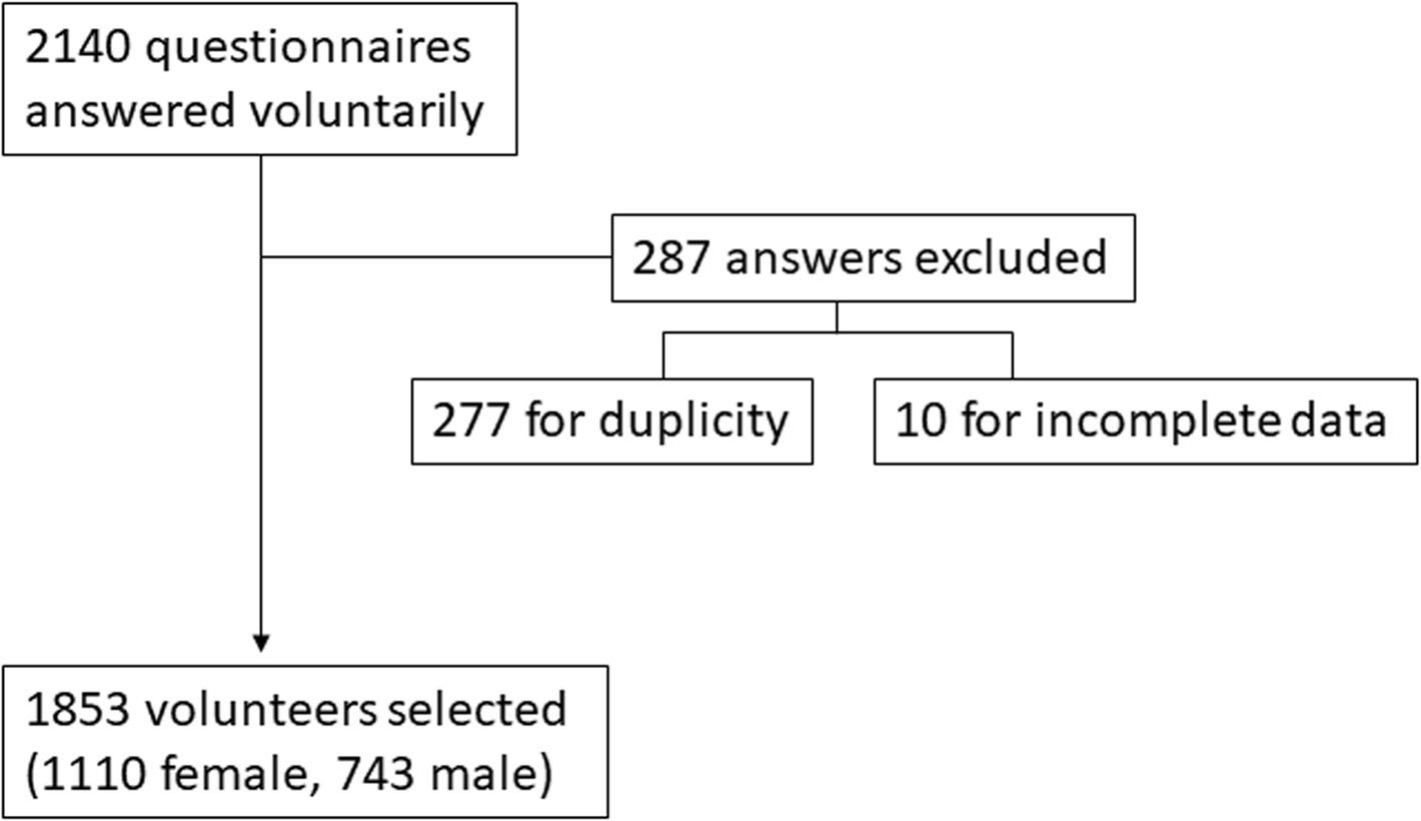
Flowchart of the study

A total of 2140 questionnaires were answered voluntarily, however, 287 were excluded because they were incomplete (10 answers) or duplicate (277 answers), which was verified considering the e-mail address reported, totalling 1853 (1110 female and 743 male) participants of the study, as shown in Fig. 1.

### Ethics approval and consent to participate

The study was approved by the Human Research Ethics Committee of the Federal University of São Paulo UNIFESP (Approval number: 4.073.442) and conformed to the principles outlined in the Declaration of Helsinki. Before responding to the survey, the volunteers read and agreed to the informed written consent. If they agreed to participate in the study, the volunteers provided the e-mail address, which was used to verify duplicity in responses.

### Statistical analysis

According to the Kolmogorov-Smirnov test, no variables presented a normal distribution. Variables were expressed as median and interquartile range. Categorical variables were expressed in absolute numbers and/or percentages. The Mann-Whitney test was used to verify differences between sexes according to age, body mass and height. The measurements of the effect size were calculated by dividing the mean difference by the standard deviation. The magnitude of the effect sizes was judged according to the following criteria: *d* = 0.2 considered a ‘small’ effect size; 0.5 represented a ‘medium’ effect size; and 0.8 a ‘large’ effect size [36]. The Wilcoxon test was used to verify differences in physical activity level between the period before the pandemic period and the current pandemic period. For comparison between sexes and family income, IPAQ at social distancing period, PHQ-9, GAD-7, and ΔIPAQ, a Chi-square test was employed. Chi-square tests were also employed in order to compare PHQ-9 and GAD-7 with IPAQ during the social distancing period, ΔIPAQ, and family income and Chi-square test was also employed to verify the association between PHQ-9 and GAD-7 levels. The Kruskal-Wallis test was used to verify age differences between each level of the PHQ-9 and GAD-7 questionnaire. The Kruskal-Wallis test was complemented by post hoc tests (pairwise analysis). Statistical analysis was performed using SPSS v 21.0 (Chicago, Illinois, USA). In all comparisons, *p* values < 5% were considered statistically significant.

## Results

Descriptive data of the participants age, body mass, height and BMI were presented in Table 1. For between sexes comparisons, male participants of the present study presented significantly higher values of age, body mass, height and BMI (Table 1).

**Table 1:** Descriptive characteristics of the participants

Variables	Whole sample ***N*** = 1843	Male *N* = 743		Female *N* = 1110	t and p value		Effect size	CI (95%)
**Age (years)**	38.6 ± 12.4	39.7 ± 12.2		37.9 ± 12.4*	t (1,851) = 3.00 *p* < 0.001		*d* = 0.14	0.07 to 0.21
**Body mass (kg)**	71.4 ± 14.3	81.0 ± 12.6		65.0 ± 11.5*	t (1,842) = 28.17 p < 0.001		*d* = 1.32	1.00 to 1.60
**Height (cm)**	168.8 ± 9.3	176.8 ± 6.9		163.5 ± 6.2*	t (1,851) = 42.70 *p* = 0.003		*d* = 1.99	1.50 to 2.50
**BMI (kg/m**^**2**^**)**	24.9 ± 3.8	25.8 ± 3.2		24.3 ± 4.0*	t (1,842) =8.78 p < 0.001		*d* = 0.41	0.24 to 0.58

Values were expressed as mean ± SD. * *p* < 0.05; t (t test value); *d* (cohen’s *d*); CI - confidence interval; BMI – body mass index

The number of participants and the percentage values according to BMI, the adoption time of social distancing, the adhesion level to social distancing, family income, physical activity levels adopted pre-pandemic period and adopted at current period, the change in physical activity levels in relation to those prior to social distancing, depression and anxiety symptons were presented in Table 2. In the whole sample, most participants were eutrophic, adopted physical distancing measures for 76 to 90 days, maintained partial restriction, leaving only for essential non-work activities, received more than 11 minimum wages, were very active, did not alter their physical activity level during pandemic period, presented mild depression symptoms and no anxiety disorder. The male sample differs from the whole sample because most participants were overweight, adopted physical distancing measures for 61 to 75 days and presented minimal depression symptoms. The female sample differs from the whole sample because most participants were active and have mild anxiety disorder **(**Table 2**)**.

**Table 2:** Number of participants and percentage values for the whole sample and for each sex

Variables	Whole sample (*n* = 1853)	Male sample (*n* = 753)	Female sample (*n* = 1100)
**BMI**			
Underweight	26 (1.4%)	1 (0.1%)	25 (2.3%)
Eutrophic	920 (49.9%)	270 (36.6%)	650 (58.8%)
Overweight	691 (37.5%)	378 (51.2%)	312 (28.2%)
Obese	176 (9.5%)	79 (10.7%)	97 (8.8%)
Extremetly obese	32 (1.7%)	10 (1.4%)	22 (2.0%)
**Time of adoption**			
social distancing for less than 30 days	178 (9.6%)	96 (12.9%)	82 (7.4%)
between 30 and 45 days	177 (9.6%)	76 (10.2%)	101 (9.1%)
between 46 and 60 days	236 (12.8%)	99 (13.3%)	137 (12.4%)
between 61 and 75 days	491 (26.6%)	186 (25.0%)	305 (27.6%)
between 76 and 90 days	497 (26.9%)	178 (24%)	319 (28.8%)
for more than 91 days	270 (14.6%)	108 (14.5%)	162 (14.6%)
**Restriction level**			
completely adhered to the social distancing recommendations	174 (9.4%)	50 (6.7%)	124 (11.2%)
maintained partial restriction, leaving only for essential non-work activities	970 (52.4%)	366 (49.4%)	604 (54.4%)
maintained partial restriction, leaving only for essential activities including work activities	604 (32.6%)	251 (33.9%)	353 (31.8%)
did not adhere to the social distancing recommendations	103 (5.6%)	74 (10.0%)	29 (2.6%)
**Family income**			
less than 1 minimum wage	43 (2.3%)	14 (1.9%)	29 (2.6%)
between 1 and 2 minimum wages	89 (4.8%)	26 (3.5%)	63 (5.7%)
receive between 3 and 6 minimum wages	664 (35.8%)	274 (36.9%)	390 (35.1%)
between 7 and 10 minimum wages	345 (18.6%)	129 (17.4%)	216 (19.5%)
more than 11 minimum wages	712 (38.4%)	300 (40.4%)	412 (37.1%)
**IPAQ current**			
not active	85 (4.6%)	27 (3.6%)	58 (5.2%)
irregularly active B	209 (11.3%)	67 (9.0%)	142 (12.8%)
irregularly active A	141 (7.6%)	54 (7.3%)	87 (7.8%)
active	693 (37.4%)	256 (34.5%)	437 (39.4%)
Very active	725 (39.1%)	339 (45.6%)	386 (34.8%)
**IPAQ pre-pandemic**			
not active	31 (1.7%)	5 (0.7%)	26 (2.3%)
irregularly active B	70 (3.8%)	26 (3.5%)	49 (4.0%)
irregularly active A	75 (4%)	20 (2.7%)	55 (5.0%)
active	519 (28%)	181 (24.4%)	338 (30.5%)
Very active	1158 (62.5%)	511 (68.8%)	647 (58.3%)
**Δ IPAQ**			
reduced their physical activity level	684 (36.9%)	250 (33.6%)	434 (39.1%)
did not alter their physical activity level	1051 (56.7%)	454 (61.1%)	597 (53.8%)
increased their physical activity level	118 (6.4%)	39 (5.2%)	79 (7.1%)
**PHQ-9**			
minimal depression	635 (34,3%)	345 (46.4%)	290 (26.1%)
mild depression	670 (36,2%)	342 (32.6%)	428 (38.6%)
moderate depression	304 (16,4%)	95 (12.8%)	209 (18.8%)
moderately severe depression	155 (8,4%)	37 (5.0%)	118 (10.6%)
severe depression	89 (4,8%)	24 (3.2%)	65 (5.9%)
**GAD-7**			
no anxiety disorder	750 (40,5%)	378 (50.9%)	372 (33.5%)
have mild anxiety	674 (36,4%)	250 (33.6%)	424 (38.2%)
moderate anxiety	265 (14.3%)	77 (10.4%)	188 (16.9%)
have a severe anxiety disorder	164 (8.9%)	38 (5.1%)	126 (11.4%)

Number of participants (percentage values); *BMI* body mass index, *IPAQ* International physical activity questionnaire, *PHQ-9* Patient Health Questionnaire-9; Δ IPAQ - the difference between the current and pre-pandemic categories of IPAQ

The association between mood state (depression and anxiety level) with the dependent variables was studied and there were significant associations between depression level (PHQ-9) and family monthly income [the higher adjusted residual was 7.2 for PHQ-9 classified as 4 (severe depression) and family income classified as 0 (less than 1 minimum wage)], level of restriction adopted during the pandemic period [the higher adjusted residual was 4.4 for PHQ-9 classified as 0 (minimal depression) and restriction level classified as 3 (not taking any measures of social distancing)], current IPAQ (the higher adjusted residual was 5.2 for PHQ-9 classified as 0 (minimal depression) and IPAQ level classified as 4 (very active), and Δ IPAQ (the higher adjusted residual was 5.3 for PHQ-9 classified as 0 (minimal depression) and Δ IPAQ classified as 0 (do not change the physical activity level), but not regarding the period for which social distancing measures were adopted. These data were analyzed using the Chi-square test, and the results are shown in Table 3.

**Table 3:** Chi-square test of association between PHQ-9 and analyzed variables

Variables		Df	p value	Cramér’s V
**Time of adoption**	19.97	20	0.46	0.05
**Restriction level**	33.28	12	< 0.001*	0.07
**Family income**	104.95	16	< 0.001*	0.12
**IPAQ current**	88.82	16	< 0.001*	0.11
**Δ IPAQ**	61.89	8	< 0.001*	0.18

* Statistically significant association (*p* ≤ 0.05); IPAQ - International physical activity questionnaire; PHQ-9 - Patient Health Questionnaire-9; Δ IPAQ - the difference between the current and pre-pandemic categories of IPAQ; X^2^ –Chi-square result; df – degrees of freedom

Additionally, age differences regarding each depression level (PHQ-9) were investigated using the Kruskal-Wallis test. The results showed a significant difference between age and PHQ-9 categories [H(4) = 214.5; *p* < 0.001]. The age [43 (19) years old] of the responders who have no depression was significantly older than that of those who were moderately depressive [34 (14) years old], which was also older than the median age of those who were severely depressive [30 (15 years old)].

There were significant associations between anxiety level (GAD7) and family monthly income (the higher adjusted residual was 5.0 for GAD-7 classified as 3 (severe anxiety disorder) and family income classified as 0 (less than 1 minimum wage), level of restriction adopted during the COVID-19 pandemic period (the higher adjusted residual was 2.8 for GAD-7 classified as 0 (no anxiety disorder) and restriction level classified as 3 (not taking any measures of social distancing), current IPAQ (the higher adjusted residual was 3.1 for GAD-7 classified as 0 (no anxiety disorder) and IPAQ level classified as 4 (very active), and Δ IPAQ (the higher adjusted residual was 3.4 for GAD-7 classified as 0 (no anxiety disorder) and Δ IPAQ level classified as 0 (do not change the physical activity level), but not regarding the period which social distancing measures were adopted. These data were analyzed using the Chi-square test, and the results are shown in Table 4.

**Table 4:** Chi-square test of association between GAD-7 and analyzed variables

Variables		Df	p value	Cramér’s V
**Time of adoption**	14.49	15	0.48	0.05
**Restriction level**	21.96	9	0.009*	0.06
**Family income**	50.50	12	< 0.001*	0.09
**IPAQ current**	35.09	12	< 0.001*	0.08
**Δ IPAQ**	36.99	6	< 0.001*	0.14

* Statistically significant association (*p* ≤ 0.05); *IPAQ* International physical activity questionnaire, *GAD-7-* General Anxiety Disorder-7; Δ IPAQ - the difference between the current and pre-pandemic categories of IPAQ; X^2^ – Chi-square result; df – degrees of freedom

Age differences regarding each anxiety level (GAD-7) were investigated via the Kruskal Wallis test. The results also showed a significant difference between the age of the different anxiety groups (N (3) = 176.4; p < 0.001). The age of the responders who had no anxiety disorder [42 (30) years old] was significantly higher than that of those who were mild anxiety [35 (15)] years old), which was also higher than the age of those who presented severe anxiety disorder [31.5 (13) years old].

The Chi-square test also has been used to verify the association level between depression and anxiety levels. The results showed a significant association between them (= 1463, Df = 12, *p*-value < 0.001 and Cramér’s V = 0.513).

There were significant association between sex (male or female) and BMI [the category that present the largest adjusted residuals (10.0 for males) was the category 3 (overweight)], the time of adoption restriction measurements [the category that present the largest adjusted residuals (3.9 for males) was the category 0 (less than 30 days)], the level of restriction adopted during the pandemic [the category that present the largest adjusted residuals (6.8 for males) was the category 3 (not taking any measures of social distancing)], current IPAQ values [the category that present the largest adjusted residuals (4.7 for males) was the category 4 (very active)], pre-pandemic IPAQ values [the category that present the largest adjusted residuals (4.6 for males) was the category 4 (very active)], Δ IPAQ [the category that present the largest adjusted residuals (− 2.4 for males) was the category 0 (decrease the physical activity level)], PHQ-9 score [the category that present the largest adjusted residuals (9.0 for males) was the category 0 (minimal depression)], and GAD-7 score [the category that present the largest adjusted residuals (7.5 for males) was the category 0 (no anxiety disorder)]. There were no significant association between sex (male or female) and family monthly income observed. These data were analyzed using the Chi-square test, and the results are shown in Table 5.

**Table 5:** Chi-square test of association between sex and analyzed variables

Variables		Df	p value	Cramér’s V
**BMI**	123.23	4	< 0.001*	0.26
**Time of adoption**	19.89	5	0.01	0.10
**Restriction level**	55.39	3	< 0.001*	0.17
**Family income**	8.07	4	0.09	0.06
**IPAQ current**	24.54	4	< 0.001*	0.11
**IPAQ pre-pandemic**	27.03	4	< 0.001*	0.12
**Δ IPAQ**	10.22	2	0.006*	0.07
**PHQ-9**	91.26	4	< 0.001*	0.22
**GAD-7**	68.69	3	< 0.001*	0.19

* Statistically significant association (*p* ≤ 0.05); BMI – body mass index; IPAQ - International physical activity questionnaire; PHQ-9 - Patient Health Questionnaire-9; GAD-7- General Anxiety Disorder-7; Δ IPAQ - the difference between the current and pre-pandemic categories of IPAQ; X^2^ – Chi-square result; df – degrees of freedom

## Discussion

The main findings from the present study were: (i) the physical activity level adopted during the period of social distancing was significantly lower than that prior to this period, (ii) about 30% of the respondents presented moderate or severe symptoms of depression, and around 23.3% showed moderate or severe symptoms of anxiety during the social distancing period, (iii) the depression and anxiety scores were significantly associated, (iv) low levels of physical activity, low family monthly income and the participants’ age were associated with higher incidences of anxiety and depression, (v) there was more frequency of individuals than the expected who did not alter their physical activity level after the adoption of social distancing experiencing lower levels of depression and anxiety and, (vi) there was a higher frequency of men than of women who were very active, who did not change their physical activity level during the social distancing period and who had no symptoms of depression and anxiety.

The level of physical activity was significantly reduced during the social distancing period. Prior to the COVID-19 pandemic period, 69% of the volunteers (83% male and 46% female) were classified as very active, and during the social distancing period, this percentage dropped to 39% (50% male and 31% female). In Italy, were more strict rules of social distancing were adopted, including lockdown, an important reduction of physical activity was also observed [37]. To be classified as a very active person, it is necessary to perform at least 30 min of vigorous activity 5 times a week (or 20 min of vigorous activity 3 times a week plus 30 min of moderate activity 5 times a week) (IPAQ) [31]. This physical activity level has been associated with several healthcare benefits, including a lower risk of cardiovascular morbidity and mortality [38]. On the other hand, a lower physical activity level due to sustained social distancing potentially increases the risk of damaging the immune, respiratory, cardiovascular, musculoskeletal systems as well as compromising mental health [7]. This known damage from low physical activity can be especially harmful during this pandemic period. Although the SARS-CoV-2 usually first compromises the functioning of the lungs, it can also infect almost all major organs in the body [39]. Therefore superior cardiorespiratory conditioning should also help to combat the disease. Strong respiratory muscles and aerobic conditioning may help individuals who develop COVID-19 and require ventilator support, mainly during the ventilator weaning process [7]. Regarding the sex difference for physical activity, the results showed that the men presented higher physical activity levels, mainly in very activity domain, which also was found by Oyeyemi et al. [40]. Indeed, during the pandemic, men and women presented a decrease in physical activity levels; however, the difference between sex remain. Prior to the pandemic period, 9.5% of the participants were classified as insufficiently active (A and B) or inactive people and this number increased to 23% after the COVID-19 pandemic. There was a 147% increase in insufficiently active (A and B) or inactive people (males increased 190%, and inactive females increased 129%). This result is concerning because physical inactivity was classified by the World Health Organization (WHO) [41] as the fourth leading risk factor for global mortality, and there is recent evidence suggesting that a sedentary lifestyle is independently associated with cardiovascular diseases [42].

Another worrying result found in the present study concerned the incidences of symptoms related to depression and anxiety. Thirty percent of the total sample (20% male and 34% female) presented moderate or severe depression symptoms, and 23% of the total sample (15% male and 27% female) presented symptoms of moderate or severe anxiety. It is interesting to note that there is also a significant association between the presence of anxiety and depression symptoms, as has also been previously demonstrated that approximately 50% of people who present mood health disorder meet criteria for both depression and anxiety [43, 44]. Cao et al. [45] and Gao et al. [46] also found similar percentages for cases of anxiety (22.6 and 22.4%, respectively) for a Chinese sample of both sexes. On the other hand, Choi et al. [47] demonstrated that 19% suffered from depression, and 14% from anxiety in a cross-sectional study conducted in Hong Kong. Puccinelli et al. [48] demonstrated in a paper presenting preliminary results that 22.8% of the Brazilians and 7% of the Swiss presented moderate or severe depression symptons. The same criteria for depression and anxiety were used in the above studies (PHQ-9 score ≥ 10 and GAD-7 score ≥ 10). In addition to the COVID-19 pandemic, Brazil’s president, Jair Bolsonaro, continues to discourage physical distancing measures along with the use of face masks, contrary to the recommendations of health organizations [49]. This has led to an increased sense of insecurity and anxiety amongst the Brazilian population regarding the COVID-19 disease [50]. Moreover, the political and economic instability that the country is undergoing may also be contributing to the high incidences of depression and anxiety. Beyond the pandemic, according to WHO, the prevalence of depression and anxiety is highest than world prevalence (5.8 and 9.3%, respectively while world prevalence is 4.4 and 3.6%, respectively) [51]. As such, Brazil has one of the world highest prevalence of depression and anxiety.

Indeed, there is a significant association between both anxiety and depression and physical activity. Thre are a higher frequency than the expected of very active people presenting minimum depression symptoms and no anxiety disorder.

In the current study, the importance of physical activity related to mental health, the difference in physical activity levels between the pre-pandemic and physical distancing (current period) was also assessed. Those who did not alter their level of activity, and therefore managed to remain active in some way, reported a higher frequency of lower depression and anxiety symptoms. These findings reinforce the importance to find ways to increase physical activity level. In this context, home-based exercise programs and stimuli to interrupt physical inactivity and sedentary behaviour, resulting from the necessary confinement policies to contain the spread of SARS-CoV-2 could be a feasible option, mainly when the most Brazilian cities parks, gym and sport clubs are closed. There are some useful tips for Home-Based Physical Activity suggested by Ricci et al. [52], Souza et al. [53] and by Viana & de Lira [54], such as taking active short breaks, walking, following online exercise classes, playing with children or guiding the elderly to stay active.

One of the factors associated with PHQ-9 and GAD-7 scores are the level of physical distancing level adopted by participants. Participants who did not adhere to physical distancing recommendations presented a higher frequency than the expected of minimal depression symptoms and no anxiety disorders, suggesting that physical distancing affected mental health. Another factor studied was the family monthly income. A significant result to note was that there was a higher frequency than the expected of individuals who receive less than one minimum wage (which corresponds to less than 200 American dollars per month) presenting severe depression (adjusted residuals 7.2) or anxiety (adjusted residuals 5.0). This situation is very worrying because the necessary physical distancing measures not only have an impact on health but also can result in a devastating threat to economy, which may reduce a family’s income even further. The unemployment situation and the lack of prospects of returning to work are other factors that can have a negative impact on mental health [46, 55]. In a previous systematic review, Vindegaard & Eriksen Benros [56] also pointed out the importance of steady family income to preserve mental health. Interestingly enough, the period of time for which an individual is in social distancing has not impacted mental health. It is possible that there is a ceiling effect, that is, social distancing has a negative impact on depression or anxiety symptoms, but that more time of isolation does not further worsen these symptoms.

Finally, there is also a significant difference between age groups, according to the PHQ-9 and GAD-7 questionnaire. Younger respondents presented more symptoms of depression than the older ones. In relation to anxiety levels, younger respondents were also found to be more anxious than older ones. Gao et al. [46] also evaluated people between 18 to 85 years old, and the authors also found a higher incidence of depression among those between 21 and 30 years of age. One possible reason may be that increased anxiety, and depression symptoms among young people are due to their higher social media exposition, one of the main channels used for updating COVID-19 information [46, 57]. However, considering age as a risk factor for depression and anxiety provided inconsistent data, given that the elderly (over 60 years of age) also presented high levels of these mental illnesses [55].

Regarding sex differences in relation to depression and anxiety levels, the results showed that women presented a higher frequency of depression and anxiety. This had already been demonstrated in studies of Chinese and Italian populations [55, 58]. Furthermore, according to WHO, women present a higher prevalence of mood disorders than men in all world regions [51]. There is also a higher frequency of male participants who were very physically active and a higher frequency of males who did not change their physical activity level during the social distancing period. There was a higher frequency of male participants who did not adhere to the social distancing recommendations, which may be a contributing factor to their lower frequency of anxiety and depression. However, the design of the present study design does not allow us to affirm if there is a causal relationship between these factors.

As a cross-sectional study, a limitation of the present study is the lack of anxiety and depression assessment before the pandemic period, moreover it is not possible to establish causal relationships between variables, as they were measured at the same time. In addition, it was also necessary for volunteers to remember when they were answering the questionnaire (June 2020) what was their physical activity level before the pandemic period (March 2020). Another possible bias is concerning the sample evaluated. Despite having1,853 respondents, the study was also disseminated via e-mail and social networks, which may not be representative of the entire population of the country, but only of people who have access to the internet and that use social networks. Finally, in June 2020, there were a series of government guidelines for people not to leave their homes. The restaurants, parks, sports clubs and shops were all closed. It is possible that when the volunteers were asked about the level of restriction they were adopting, they became embarrassed to answer the truth if they were not following the government’s recommendations.

## Conclusion

Considering the dramatic change in lifestyle linked to physical inactivity and all non-communicable diseases associated with this condition, such as diabetes, cardiovascular disease and obesity as well as the significant association between physical inactivity and mental diseases, it is clear that people during this social distancing period are becoming much more physically and mentally vulnerable, which affects their ability to combat a possible COVID-19 infection. Therefore, physical activity programs should be encouraged, given that they respect the required social distancing to contain the spread of SARS-CoV-2.

## Data Availability

The datasets used and/or analyzed during the current study are available from the corresponding author on reasonable request.

## Abbreviations

PHQ-9: Patient Health Questionnaire-9
GAD-7: General Anxiety Disorder-7
IPAQ: International Physical Activity Questionnaire
