# Correction to: Reduced level of physical activity during COVID-19 pandemic is associated with depression and anxiety levels: an internet-based survey

**DOI:** 10.1186/s12889-021-10684-1

**Published:** 2021-03-29

**Authors:** Paulo José Puccinelli, Taline Santos da Costa, Aldo Seffrin, Claudio Andre Barbosa de Lira, Rodrigo Luiz Vancini, Pantelis T. Nikolaidis, Beat Knechtle, Thomas Rosemann, Lee Hill, Marilia Santos Andrade

**Affiliations:** 1 Department of Physiology, Federal University of São Paulo, São Paulo, Brazil; 2 Human and Exercise Physiology Division, Faculty of Physical Education and Dance, Federal University of Goiás, Goiânia, Brazil; 3 Center for Physical Education and Sports, Federal University of Espírito Santo, Victoria, Brazil; 4 School of Health and Caring Sciences, University of West Attica, Athens, Greece; 5 Medbase St. Gallen Am Vadianplatz, St. Gallen, Switzerland; 6 Institute of Primary Care, University of Zurich, Zurich, Switzerland; 7 Divison of Gastroenterology and Nutrition, Department of Pediatrics, McMaster University, Hamilton, Canada

**Correction to: BMC Public Health (2021) 21:425**

**https://doi.org/10.1186/s12889-021-10470-z**

It was highlighted that in the original article [1], in the first sentence of the Results section of the Abstract, the numbers were interchanged. This Correction article shows the incorrect and correct sentence in the Results section of the Abstract. The original article has been updated.

**Incorrect sentence**

Results: The physical activity level adopted during the period of social distancing (3.5 ± 0.8) was lower than that the adopted prior to the pandemic period (2.9 ± 1.1, *p* < 0.001).

**Correct sentence**

Results: The physical activity level adopted during the period of social distancing (2.9 ± 1.1) was lower than that adopted prior to the pandemic period (3.5 ± 0.8, *p* <0.001).
